# Thymic hyperplasia is accurate to detect new-onset Graves’ hyperthyroidism and resolves after restoring euthyroidism

**DOI:** 10.1007/s40618-024-02355-w

**Published:** 2024-03-30

**Authors:** L. Scappaticcio, P. Caruso, N. Di Martino, P. Ferrazzano, A. Clemente, M. I. Maiorino, A. Reginelli, G. Docimo, P. F. Rambaldi, G. Bellastella, P. Trimboli, S. Cappabianca, K. Esposito

**Affiliations:** 1 Unit of Endocrinology and Metabolic Diseases, AOU University of Campania “Luigi Vanvitelli”, 80138 Naples, Italy; 2 Department of Advanced Medical and Surgical Sciences, University of Campania “Luigi Vanvitelli”, Naples, Italy; 3 Radiology and Radiotherapy Unit, Department of Precision Medicine, University of Campania “L. Vanvitelli”, Naples, Italy; 4 Division of Thyroid Surgery, AOU University of Campania “Luigi Vanvitelli”, Naples, Italy; 5 Clinic of Endocrinology and Diabetology, Lugano and Mendrisio Regional Hospital, Ente Ospedaliero Cantonale, Bellinzona, Switzerland; 6 Faculty of Biomedical Sciences, Università della Svizzera Italiana, Lugano, Switzerland

**Keywords:** Thymic hyperplasia, Graves’ disease, Ultrasound

## Abstract

**Purpose:**

Abnormal liver blood tests (ALBTs), neutropenia (NEU) and thymic hyperplasia (TH) are new features of Graves' disease (GD). Our objectives were: (a) to calculate the accuracy of TH in discriminating between Graves’ and non-Graves’ thyrotoxicosis, compared to ALBTs, NEU and Graves’ orbitopathy (GO); (b) to explore the outcome of GD-associated TH and non-GD-associated TH.

**Methods:**

We prospectively analyzed consecutive adult patients with newly diagnosed thyrotoxicosis from January 2018 to June 2023. TH was detected via neck ultrasound (nUS) then confirmed and followed by magnetic resonance imaging (MRI). For GD vs non-GD clinical sensitivity (SE) and specificity (SPEC), accuracy, positive predictive value (PPV) and negative predictive value (NPV) of GO, TH, ALBTs and NEU were calculated.

**Results:**

264 thyrotoxic patients were included. TH was found in 16.4% (20/122) of GD vs 1.4% (2/142) in non-GD (*p* < 0.001). SE, SPEC, accuracy, PPV and NPV of the four extrathyroidal manifestations of GD were as follows, respectively: GO 26%, 100%, 66%, 100%, 61%; ALBTs 41%, 89%, 69%, 76%, 66%; NEU 5%, 100%, 56%, 100%, 55%; TH 16%, 98%, 61%, 91%, 98%. In 18 of them, TH regressed within 12 months after achieving euthyroidism under anti-thyroid drug therapy, while in the remaining 2, TH regressed 6 months after thyroid surgery. In the two non-GD patients with TH, thymus disappeared along with euthyroidism.

**Conclusions:**

TH in the hyperthyroidism scenario provides a high PPV for GD. A conservative approach for the diagnostic work‐up and initial management of thyrotoxicosis-associated TH should be adopted.

## Introduction

Clinical phenotype is currently reported to be milder than in the past, with goiter, Graves’ orbitopathy (GO) and severe excess of thyroid hormones being less frequent to date [1–3]. Therefore, due to the absence of the pathognomonic features such as GO and thyroid function testing, it may become challenging to distinguish Graves’ hyperthyroidism from other forms of thyrotoxicosis [4, 5]. For this reason, in hyperthyroidism scenario, other extrathyroidal manifestations of GD should be investigated in combination with reference laboratory [i.e., thyrotropin (TSH) receptor antibodies (TRAb)] and imaging tests (i.e., scintigraphy) to support the clinical diagnosis of GD [6]. Among the main emerging extrathyroidal manifestations of GD, we have abnormal liver blood tests (ALBTs) [7] and neutropenia (NEU) [8]. It has been proved that both ALBTs and neutropenia are quite common in untreated and new-onset GD patients, they do not usually represent a contraindication to anti-thyroid therapy (ATD) and they typically resolve while treating hyperthyroidism [7, 8].

Thymus hyperplasia (TH) refers to gross, diffuse, and symmetric enlargement of the thymus [9]. The association between GD and TH is quite known to thyroidologists but it remains largely unrecognized in clinical practice of other physicians [9]. This is probably the consequence of limited thoracic radiological evaluations for most patients with hyperthyroidism as well as a lack of awareness of this association. The association between GD and TH was first described in 1912 [10] and has been reported numerous times thereafter mostly as case reports and case series [11–15]. Thymic hyperplasia in GD is commonly discovered incidentally by chest X‐ ray or computerized tomography (CT) for other reasons [11]. It is proposed to control hyperthyroidism to improve and lead to disappearance of TH [9]. Yet, biopsy and thymectomy are recommended to be deferred in the setting of thyrotoxicosis to avoid triggering a thyroid storm [16] and should be reconsidered if the thymus fails to regress with treatment to rule out malignancy [9, 17].

Therefore, the knowledge of the relationship between these emerging extrathyroidal manifestations (i.e., ALBTs, neutropenia, TH) and hyperthyroidism is of paramount importance for proper diagnosis and therapeutic decision-making.

This study is designed to bring more light into the thyrotoxicosis-associated TH, and our specific objectives were (a) to calculate the prevalence of radiological TH in a cohort of unselected patients with thyrotoxicosis; (b) to calculate the accuracy of TH in discriminating between Graves’ and non-Graves’ thyrotoxicosis, compared to ALBTs, NEU and Graves’ orbitopathy (GO); (c) explore the outcome of GD and non-GD-associated TH.

## Materials and methods

### Study design and patients

In the current study, the Standards for Reporting Diagnostic Accuracy (STARD) statement was followed [18]. We prospectively examined consecutive adult patients with newly diagnosed and untreated thyrotoxicosis at our academic center from January 2018 to July 2023. Patients were being referred to our multidisciplinary team as they developed clinical manifestations of thyrotoxicosis (mainly cardiovascular and neurological symptoms) or, more rarely, to investigate incidental subnormal TSH. It needs to be specified that in our center patients with thyrotoxicosis routinely underwent baseline liver blood tests (LBTs) [i.e., alanine transaminase (ALT), Ɣ-glutamyl transferase (GGT), total bilirubin (BIL)], absolute neutrophil count (ANC), thyrotropin receptor antibodies (TRAb) measurement, neck ultrasonography (nUS), and thyroid scintigraphy in the same day or within few days (few weeks at the most) from the first examination.

Biochemical diagnosis of thyrotoxicosis/hyperthyroidism was based on TSH < 0.1 mIU/L with normal (subclinical) or elevated thyroid free thyroxine (fT4) and/or free triiodothyronine (fT3) alone (overt). Diagnosis of GD was confirmed by thyroid scintigraphy based on diffuse and homogeneous thyroid overactivity and reduced uptake in major salivary glands. In our center, visits were planned every 3–6 months, and clinical examination was also oriented to find extrathyroidal manifestations [i.e., GO, Graves’ dermopathy (GDerm), ALBTs, NEU and TH]. Activity and severity of GO were assessed according to the evidence-based recommendations of current guidelines [19]. Initial and ongoing therapies followed the current guidelines and recommendations [4] and patient preferences. In all patients with GD, medical therapy represented the first-line treatment for at least 18 months, and the starting dose of anti-thyroid drug (ATD) therapy (i.e., namely methimazole which was the preferred initial therapy) was established according to fT4 levels to promptly reach and stably maintain euthyroidism. Dose adjustment of ATD was pursued at every outpatient visit and controlled hyperthyroidism was defined by both fT4 and fT3 in normal ranges [4]. After 18 months of ATD therapy, medical therapy was continued for other 12–24 months or a definitive therapy (i.e., radioiodine or surgery) was adopted [4]. The Human Research Ethics Committee at the AOU University of Campania "Luigi Vanvitelli" (Naples, Italy) approved the study, and written informed consent was obtained from each study participant (Campania 2, 0003042/i).

The inclusion criteria were (a) follow-up of at least 18 months; (b) detection of TH via nUS then confirmed and followed every 6 months also by magnetic resonance imaging (MRI) until disappearance. The exclusion criteria were (a) concomitant liver disease/dysfunction; (b) preexisting diseases/drugs influencing the ANC; (c) incomplete diagnostic and follow-up test results; (d) previous history of thyroid disease or use of levothyroxine; (e) pregnancy; (f) uncontrolled hyperthyroidism at the 6-month follow-up under ATD therapy.

### Laboratory tests

Thyroid function (i.e., TSH, fT3, fT4, fT3/ fT4 ratio) and antibodies [i.e., TRAb, anti-thyroglobulin (TgAb), anti-thyroid peroxidase (TPOAb)] was assessed with immunoassay method in automated platform (Elecsys^®^ e801 Roche Diagnostics). TSH assay had analytical sensitivity of 0.001 mIU/L, and reference range was between 0.27 and 4.2 mIU/L. Normal reference ranges for fT3 and fT4 were 3.1–6.8 pmol/L and 12–22 pmol/L, respectively. The lower cut-off of TRAb for a positive sample was > 1.7 IU/L. TRAb values exceeding 40 IU/L were reported by the laboratory as > 40 IU/L. In GD patients TRAb measurement was performed after 12 months of ATD therapy and in GD patients with TH every 6 months thereafter. TgAb and TPOAb were only assessed at baseline. The lower cut-off of TgAb and TPOAb for a positive sample was > 115 IU/mL and > 35 IU/mL, respectively.

Among LBTs, ALT was determined through kinetic ultraviolet methodology and values over 31U/L were considered high; GGT was measured through kinetic colorimetric methodology and values over 38U/L were considered abnormal; BIL was determined through a colorimetric method and values over 1.2 mg/dL were considered high. ALBTs condition was defined by the presence of at least one abnormal LBT among ALT, GGT and BIL. LBTs were assessed at each visit under ATD therapy [4].

Measurement of ANC was determined through colorimetric test (Siemens Advia 2120) and neutropenia was defined as an ANC ≤ 1.8 × 10^9^/L. Baseline diagnostic and follow-up laboratory results were extracted for the present study. ANC was assessed at each visit under ATD therapy [4].

### Neck ultrasonography

nUS was performed by an experienced thyroidologist (LS) through an ultrasound device (MyLab™Six, Esaote) with a 7–14 MHz wide band linear transducer. The color gain was adjusted, so that artefacts were prevented. nUS was performed at each visit. Specifically, the ultrasound examination of thyroid vascularity (qualitative assessment) and volume, along with nodules and cervical lymph nodes detection and characterization, were systematically conducted.

In our study, nUS aimed to explore the presence of TH (i.e., cervical portion) and served to request MRI examination. On nUS TH appeared as a cervical mass located next clavicular fossa with partial immersion in superior mediastinum. TH typically had trapezoidal shape, overall decreased echogenicity (compared to thyroid), heterogeneous “marbled” echo pattern and reduced/nearly absent vascularity (see Fig. 2 below).

### Magnetic resonance imaging

All MRI examinations were performed on 1.5 Tesla MRI scanner (GE Signa Voyager HD, GE Healthcare, Milwaukee, WI, USA) by using a phased-array body coil centered on the mediastinum before and after the intravenous injection of paramagnetic contrast medium (Gadobutrol, 0.1 mL/kg, Gadovist^®^, Schering AG, Berlin, Germany). The imaging protocol was chosen following the most recent recommendations in thoracic MRI imaging reports [20, 21]. Our imaging protocol included: (1) axial thin Sect. (3 mm) T2-weighted single shot fast spin-echo sequence, (2) axial thin Sect. (3 mm) T2-weighted with fat-suppression (STIR), (3) axial thin Sect. (3 mm) T1-weighted dual-echo in- and opposed-phase; (4) axial diffusion-weighted (b-values 0, 100, 800 s/mm^2^) and relative apparent diffusion coefficient (ADC) maps; (5) precontrast axial T1-weighted fast gradient-echo; (6) post-contrast axial T1-weighted fast gradient-echo (acquisition at 20–30 s, 60–70 s, 3-min and 5-min delays); (7) sagittal and coronal T1-weighted fast gradient-echo acquisitions between 3 and 5 min. Thoracic MRI examinations were performed to confirm and further assess TH initially detected on nUS. MRI aimed to calculate volume values of TH and the interval time of TH disappearance. In thyrotoxic patients with TH, MRI was performed at diagnosis (when TH was first detected on nUS) and every 6 months to follow gradual remission of TH under euthyroidism. When disappearance of TH was recorded, MRI was not repeated. If volume of TH did not reduce at the 6-month follow-up under euthyroidism, other causes of TH would be investigated (i.e., neurological, rheumatological, dermatological and hematological diseases) and proper algorithms for other conditions would be followed through consultations. All MRI examinations were evaluated by two radiologists [5- (A.C.) and 15- (A.R.) years experienced]. TH was assessed when a lobulated soft tissue mass with homogeneous appearance throughout the various MRI sequences and relatively uniform loss of signal intensity on in- and opposed-phase images was found in the mediastinal prevascular space (see Fig. 3 below).

### Thyroid scintigraphy

Fifteen minutes after intravenous administration of 185 MBq (5 mCi) of Technetium-99m (99mTc) pertechnetate, anterior images of the neck were acquired using a gamma-camera equipped with high-resolution parallel-hole collimator (Symbia^®^, Siemens) with an acquisition time of 10 min, using a 20% window centred around the 140 keV peak of 99mTc and a 128 × 128 computer matrix. Baseline scintigraphy patterns 0–2 [0, normal gland uptake; 1, decreased uptake; 2, unifocal or multifocal overactive areas with reduced or suppressed uptake in the remaining thyroid tissue, consistent with autonomously functioning thyroid nodule(*s*) (AFTN)] corresponded to non-Graves’ hyperthyroidism cases, as previously described [6].

### Statistical analysis

The quantitative variables were described as median and interquartile range and analyzed by the Mann–Whitney test, as they were not normally distributed. The dichotomous qualitative variables, on the other hand, were presented in the form of “YES”, “NO”, “Male” and “Female”, and compared with each other through the Chi-Square test. Based on the tests applied, statistical significance is obtained for *p*-values as close to 0. In our case, statistical significance is obtained for *p*-values < 0.05.

For GD vs non-GD clinical sensitivity (SE) and specificity (SPEC), accuracy, positive predictive value (PPV) and negative predictive value (NPV) of GO, TH, ALBTs and NEU were calculated based on the reference cut-off and expressed as a percentage. The graph shown (see Fig. 4 below) relates the variables "TH Volume" and "TRAb" belonging to the subgroup “GD with TH” (GD patients with TH), respectively, for the time “0”, “6”, “12”, “18”, “24” months, and it was created with the “GraphPad Prism” software, version 9.5.1 (528). All analyses were performed using the "SigmaStat" Software, version 3.5, Build 3.5.0.54, 2006.

## Results

### GD vs non-GD group

**Fig. 1:**
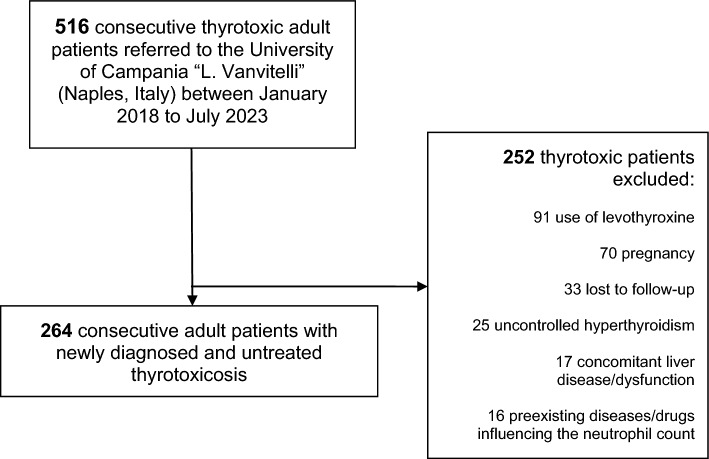
Flowchart of patients’ selection

After applying our selection criteria, we eventually included 264 untreated thyrotoxic patients (GD, *n* = 122 vs non-GD, *n* = 142) (Fig. 1). Non-GD patients included the following etiologies: 66 AFTNs, 24 toxic multinodular goiters, 22 subacute thyroiditis, 17 silent thyroiditis, 8 amiodarone-induced thyrotoxicosis, 4 Iodine-Induced hyperthyroidisms, 1 iatrogenic thyrotoxicosis secondary to thyroxine (T4) + triiodothyronine (T3) combination therapy. Several baseline characteristics (i.e., demographic, laboratory, and imaging characteristics) significantly differed between the two groups (GD vs non-GD), among which are the following: age was higher in non-GD group [57 (50–71) years vs 45 (31–53) years in GD group, *p* < 0.001]; prevalence of preexisting autoimmune diseases was higher in GD group (15% vs 1.4%, *p* 0.001); higher ALT serum levels, higher number of patients with abnormal ALT and higher ALT levels when over the cut-off in GD group [22 (15–43) U/L vs 16 (14–20) U/L in non-GD group, *p* < 0.001; 34.4% vs 8.4% in non-GD group, *p* < 0.001; 58 (43–72) U/L vs 39.5 (33–41) U/L in non-GD group, *p* 0.004]; higher GGT serum levels and higher number of patients with abnormal GGT in GD group [20 (16–34) U/L vs 16 (13–19) U/L in non-GD group, *p* < 0.001; 23% vs 1.4% in non-GD group, *p* < 0.001]; lower ANC and higher number of patients with neutropenia in GD group [3 (2.5–3.6) × 10^9^/L vs 3.7 (3.4–3.9) 10^9^/L in non-GD group, *p* < 0.001; 4.9% vs 0% in non-GD group, *p* < 0.001]; GO and GDerm were present only in GD group (26.2% and 1.6%, respectively); higher prevalence of TH in GD group (16.4% vs 1.4% in non-GD group, *p* < 0.001). Table 1 summarizes and compares the main characteristics of the two groups (GD vs non-GD).

**Table 1:** Comparison of the main characteristics of the two groups (GD vs non-GD)

Characteristics	GD (*n* = 122)	Non-GD (*n* = 142)	*p*-value
Age at diagnosis, years (IQR)	45 (31–53)	57 (50–71)	< 0.001
Females/Males (*n*)	3.36	3.44	0.947
Smoke, (yes/no)	0.49	0.39	0.497
Autoimmune diseases, *n* (%)	18 (15)	2 (1.4)	0.001
Multiple sclerosis, n	6	0	
Type 1 diabetes, n	2	0	
Vitiligo, *n*	6	0	
Psoriasis, *n*	0	2	
Thrombocytopenia, *n*	2	0	
Cutaneous lupus erythematosus, *n*	2	0	
TSH values, mIU/L (IQR)	0.004 (0.003–0.005)	0.02 (0.007–0.1)	< 0.001
fT4 values, pmol/L (IQR)	39 (31–53)	18.4 (16.9–22.4)	< 0.001
fT3 values, pmol/L (IQR)	12 (9–20)	6.1 (4.5–6.3)	< 0.001
fT3/fT4 ratio	0.31 (0.26–0.41)	0.27 (0.23–0.31)	< 0.001
TgAb, (−/ +)	1.35	4.92	< 0.001
TPOAb, (−/ +)	0.69	4.46	< 0.001
TRAb, (−/ +)	0.11	13.2	< 0.0001
ALT values, kUV (IQR)	22 (15–43)	16 (14–20)	< 0.001
ALT > cut-off, *n* (%)	42 (34)	12 (8)	< 0.001
ALT values > cut-off, kUV (IQR)	58 (43–72)	39.5 (33–41)	0.004
GGT values, kCol (IQR)	20 (16–34)	16 (13–19)	< 0.001
GGT > cut-off, *n* (%)	28 (23)	2 (1.4)	< 0.001
GGT values > cut-off, kCol (IQR)	50 (44–71)	80 (80–80)	0.196
ANC values, 10^9^/L (IQR)	3 (2.5–3.6)	3.7 (3.4–3.9)	< 0.001
NEU, *n* (%)	6 (5)	0	
ANC values ≤ cut-off, 10^9^/L (IQR)	1.6 (1.6–1.8)	0	
GO, *n* (%)	32 (26)	0	
Mild, n	24		
Moderate-severe/Severe, *n*	8		
GDerm, *n* (%)	2 (1.6)	0	
TH, *n* (%)	20 (16.4)	2 (1.4)	< 0.001

*GD* graves’ disease *IQR* interquartile range, smoke* at least 3 tobacco cigarettes/day, *TgAb* anti-thyroglobulin antibodies, TPOAb anti-thyroid peroxidase antibodies, *TRAb* thyrotropin receptor antibodies, *ALT* alanine transaminase, *GGT* γ-glutamyl transferase, *ANC* absolute neutrophil count, *NEU* neutropenia, *GO* Graves’ orbitopathy, *GDerm* Graves’ dermopathy, TH thymic hyperplasia

### Accuracy diagnostic tests of GO, ALBTs, NEU and TH

To calculate ALBTs accuracy tests, we considered that ALT and/or GGT were high overall in 57.4% (70/122) of GD vs 9.8% (14/142) in non-GD (*p*-value < 0.001). SE, SPEC, accuracy, PPV and NPV of the four extrathyroidal manifestations of GD (i.e., GO, ALBTs, NEU and TH) were as follows, respectively: GO 26%, 100%, 66%, 100%, 61%; ALBTs 41%, 89%, 69%, 76%, 66%; NEU 5%, 100%, 56%, 100%, 55%; TH 16%, 98%, 61%, 91%, 98%. Table 2 summarizes the accuracy diagnostic tests of the four extrathyroidal manifestations of GD.

**Table 2:** Accuracy diagnostic tests of the four extrathyroidal manifestations of GD

Extrathyroidal manifestation	SE	SPEC	Accuracy	PPV	NPV
GO	26	100	66	100	61
ALBTs	41	89	69	76	66
NEU	5	100	56	100	55
TH	16	98	61	91	98

*SE* sensitivity, *SPEC* specificity, *PPV* positive predictive value, *NPV* negative predictive value, *GO* Graves’ orbitopathy, *ALBTs* abnormal liver blood tests, *NEU* neutropenia, *TH* thymic hyperplasia

To calculate *ALBT*s accuracy tests we considered that alanine transaminase (*ALT*) and/or γ-glutamyl transferase (*GGT*) were high overall in 57.4% (70/122) of Graves’ disease (*GD*) vs 9.8% (14/142) in non-GD (p-value < 0.001)

### TH in the thyrotoxic context

In all 22 patients with TH MRI confirmed the presence of the thymic enlargement detected by nUS. We eventually divided the GD group in two subgroups depending on the presence of TH. We found that some baseline characteristics (i.e., demographic and laboratory characteristics) significantly differed between the two subgroups (GD without TH vs GD with TH), among which are the following: age was lower in GD with TH [(30 (25–46) years vs 48 (35–58) in GD without TH, *p* 0.002]; prevalence of preexisting autoimmune diseases was higher in GD with TH (20% vs 14%, *p* 0.001); ft4 and ft3 levels were higher in GD with TH [48 (39–53) pmol/L vs 34 (29.4–52) pmol/L, *p* 0.003, and 18.9 (14–24) pmol/L vs 11.6 (8.3–17) pmol/L, *p* 0.005, respectively]; TgAb ratio (no/yes) was lower in GD with TH (0.67 vs 1.55, *p* 0.001) and TPOAb ratio (no/yes) was higher in GD with TH (1.0 vs 0.65, *p* 0.001); higher number of patients with abnormal ALT in GD with TH subgroup (70% vs 27%, *p* 0.001). Table 3 summarizes and compares the main characteristics of the two subgroups (GD without TH vs GD with TH).

**Table 3:** Comparison of the main characteristics of the two subgroups (GD without TH vs GD with TH)

Characteristics	GD without TH (*n* = 102)	GD with TH (*n* = 20)	*p*-value
Age at diagnosis, years (IQR)	48 (35–58)	30 (25–46)	0.002
Females/Males (*n*)	3.66	2.33	0.597
Smoke, (yes/no)	0.46	0.67	0.623
Autoimmune diseases, *n* (%)	14 (14)	4 (20)	< 0.001
Multiple sclerosis, *n*	4	2	
Type 1 diabetes, *n*	2	0	
Vitiligo, *n*	4	2	
Psoriasis, *n*	0	0	
Thrombocytopenia, *n*	2	0	
Cutaneous lupus erythematosus, *n*	2	0	
TSH values, mIU/L (IQR)	0.004 (0.003–0.005)	0,004 (0.002–0.005)	0.835
fT4 values, pmol/L (IQR)	34 (29.4–52)	48 (39–53)	0.003
fT3 values, pmol/L (IQR)	11.6 (8.3–17)	18.9 (14–24)	0.005
fT3/fT4 ratio	0.31 (0.26–0.41)	0.35 (0.3–0.5)	0.07
TgAb, (−/ +)	1.55	0.67	< 0.001
TPOAb, (−/ +)	0.65	1.00	< 0.001
TRAb values, IU/L (IQR)	9.5 (6–18)	11 (6.4–16)	0.759
ALT values, kUV (IQR)	20 (15–34)	42 (16–55)	0.06
ALT > cut-off, *n* (%)	28 (27)	14 (70)	< 0.001
ALT values > cut-off, kUV (IQR)	59 (43–74)	55 (37–72)	0.370
GGT values, kCol (IQR)	20 (16–32)	27.5 (13–46)	0.591
GGT > cut-off, *n* (%)	20 (19)	8 (40)	0.091
GGT values > cut-off, kCol (IQR)	51 (44–71)	48.5 (42.5–119.5)	0.858
ANC values, 10^9^/L (IQR)	3.1 (2.5–3.7)	3.1 (2.5–3.7)	0.416
NEU, *n* (%)	6 (6)	0	
ANC values ≤ cut-off, 10^9^/L (IQR)	1.6 (1.6–1.8)	0	
GO, yes/no	0.31	0.67	0.210

*GD* Graves' disease, *TH* thymic hyperplasia, *IQR* interquartile range. Smoke* at least 3 tobacco cigarettes/day, *TgAb* anti-thyroglobulin antibodies, *TPOAb* anti-thyroid peroxidase antibodies, *TRAb* thyrotropin receptor antibodies, *ALT* alanine transaminase, *GGT* γ-glutamyl transferase, *ANC* absolute neutrophil count, *NEU* neutropenia, *GO* Graves’ orbitopathy

### GD-associated TH

**Fig. 2:**
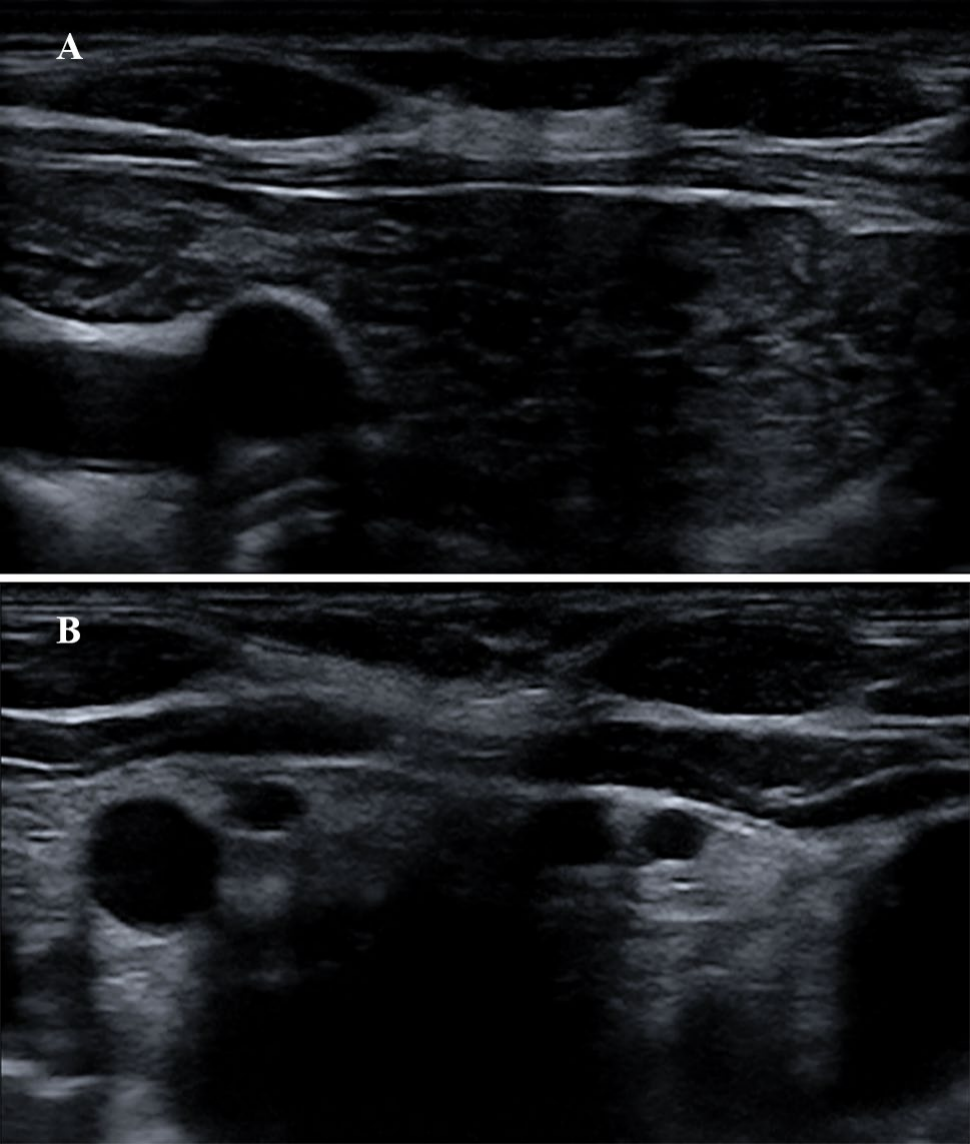
Representative case of GD-associated TH by nUS, at baseline (**a**) and after achieving stable euthyroidism (**b**). **a** 29-year-old woman with GD-associated TH: at diagnosis, thymus is sensibly enlarged in nUS transversal projection; **b** after 6 months of methimazole-based therapy under stable euthyroidism, the thymus parenchyma is not seen any more at nUS.*TH* thymic hyperplasia, *GD* Graves’ disease, *nUS* neck ultrasound

**Fig. 3:**
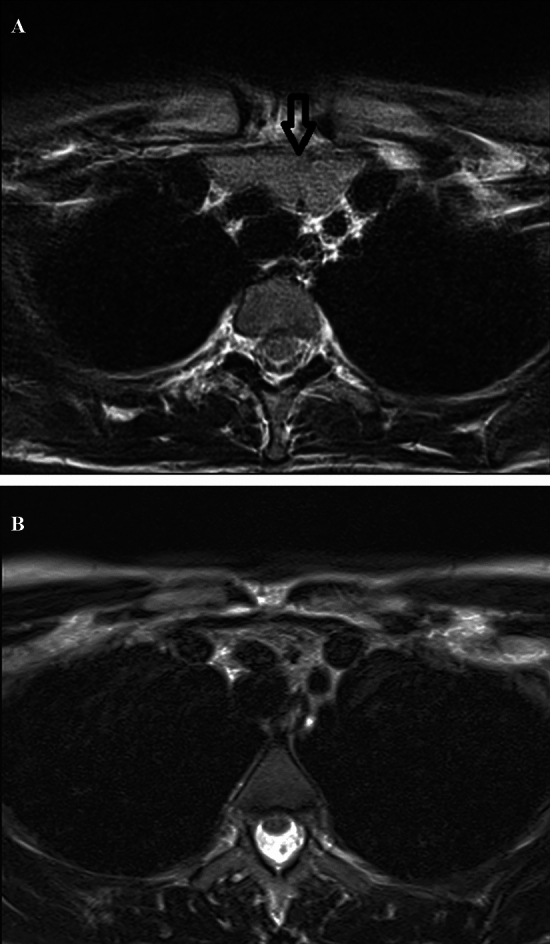
Representative case of GD-associated TH (the same patient of Fig. 2) evaluated by thoracic MRI, at baseline (**a**) and after achieving stable euthyroidism (**b**).**a** 29-year-old woman with GD-associated TH (the same patient of Fig. 2): at diagnosis, axial high-resolution T2 image demonstrated a well lobulated 50 mm (9.8 mL) soft tissue mass with homogeneous signal intensity in the mediastinal prevascular space extending superiorly to the level of the left brachiocephalic trunk (arrow), **b** that disappeared after 6 months of methimazole-based therapy under stable euthyroidism. TH, thymic hyperplasia; GD, Graves’ disease, MRI, magnetic resonance imaging

In the 20 GD patients with TH, the median volume of thymus at MRI was 8.5 (7.5–11) mL. Median time of TH disappearance both at nUS and MRI was 12 (6–12) months. At the 6-month follow-up volume reduction rate (VRR) of TH was 60% (50–70.7%) and TH was not visible in six patients. At the 12-month follow-up, TH disappeared in other 12 patients. In 18 of 20 (90%) of cases, TH totally regressed within 12 months under biochemical and clinical euthyroidism through ATD therapy. In the remaining two GD patients TH gradually regressed at the 12- and 18-month follow-up and it disappeared 6 months after thyroid surgery (the latter was adopted after 18 months of ATD therapy). Figures 2,  3 depict how TH appear at nUS and MRI, respectively, and how TH was not evident after 6 months of ATD therapy in a 29-year woman with GD.

**Fig. 4:**
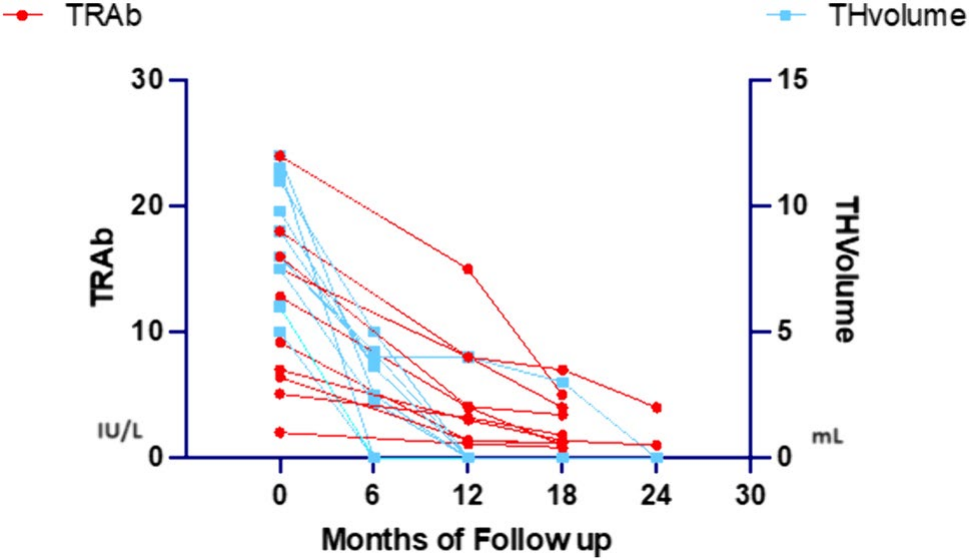
Time-dependent decreasing trend of TRAb values (IU/L) and TH volumes (mL) in the 20 GD patients with TH under therapy determining stable euthyroidism *TRAb* thyrotropin receptor antibodies, *TH* thymic hyperplasia, *GD* Graves’ disease

In ten out of the GD patients with TH TRAb disappeared (i.e., six cases and four cases at the 12-month and 18-month visits, respectively) in combination with the TH disappearance. On the contrary, in the other ten GD patients with TH TRAb did not disappear at the 18-month visit despite the disappearance of TH. Figure 4 shows the time-dependent decreasing trend of TRAb values and TH volumes in the 20 GD patients with TH under therapy determining biochemically and clinically euthyroidism.

### Non-GD-associated TH

Two non-GD patients was found to have TH. One was a 32-year-old girl with TH of 6.5 mL without comorbidities and with silent thyroiditis, TgAb and TPOAb positivity, and negative TRAb. The other case was represented by a 37-year-old girl with TH of 5.8 mL without comorbidities and iatrogenic thyrotoxicosis secondary to T4 + T3 combination therapy for hypothyroidism due to Hashimoto's thyroiditis. In both patients, TH gradually disappeared along with return to normal thyroid function as demonstrated by serial nUS examinations and the 6-month follow-up MRI. In the patient with silent thyroiditis, the involution of TH occurred along with spontaneous achievement of euthyroidism, while in the thyrotoxic patient with T4 + T3 therapy TH disappeared after switching to T4 monotherapy and achievement of biochemical euthyroidism.

## Discussion

The knowledge of the relationship between the emerging extrathyroidal manifestations and GD is of paramount importance for proper diagnosis and therapeutic decision-making [7–9]. In hyperthyroidism, thymic enlargement can be the expression of thyrotoxicosis itself, or it more rarely indicates the coexistence of an independent autoimmune disease or a cancer [9]. It is necessary to be careful that the lack of familiarity about the association of TH with GD may result in an aggressive management including surgical intervention, along with its associated risks and costs [9].

We eventually included a large cohort of new-onset and untreated thyrotoxic patients, roughly half with GD and half with non-GD thyrotoxicosis. As expected, several baseline characteristics differed between the two groups (GD vs non-GD), among which are the following: age was higher in non-GD group [4]; prevalence of preexisting autoimmune diseases was higher in GD group [22]; higher ALT and GGT serum levels and higher number of patients with abnormal ALT and/or GGT were found in GD group [7]; NEU, GO and GDerm were present only in GD patients, with prevalences consistent with that reported in the current literature (i.e., 4.9%, 26.2% and 1.6%, respectively) [1, 5, 8]. Moreover, we found 22 cases (i.e., almost 1 out 10 thyrotoxic patients) of TH initially detected by nUS and then confirmed and further explored by thoracic MRI. We had no case of TH detected by nUS which were not confirmed by thoracic MRI. The distribution of TH was different among the two groups: higher prevalence was found among GD patients (i.e., 20 cases, corresponding to more than 15% of the GD group) than in non-GD group (i.e., 2 cases, corresponding to more than 1% of the non-GD group). We expected that TH was more prevalent in the GD group, since we knew that the production of TRAb is a consequence of the impairment of self‐tolerance which occurs in the thymus [22] and that the thyroid stimulating hormone (TSH) receptor is also found in extrathyroidal human tissues, including the thymus [23, 24] where it has been shown to be functional [24, 25]. These figures lead us to claim that in the GD scenario there was a high chance to encounter thymic enlargement, while TH was exceptional but not inconceivable in non-GD thyrotoxic patients.

Compared to GO and the other extrathyroidal manifestations (i.e., ALBTs and NEU), TH was associated to a good accuracy and a high PPV in discriminating between Graves’ and non-Graves’ thyrotoxicosis. In practice, this meant that, since TH was common among GD patients (i.e., almost one out of 10 GD patients), when we found TH at nUS there was a high chance (more than 90%) that the thyrotoxic patients had GD. Therefore, in thyrotoxicosis scenario, the presence of TH could be firstly investigated by cervical US and regarded as an accurate manifestation to discriminate GD from non-GD patients. However, at nUS, the absence of TH does not exclude the presence of GD.

In patients with GD some features seemed to be important factors affecting the thymic appearance. Specifically, we found that GD patients with TH were younger, had higher prevalence of preexisting autoimmune diseases and higher ft4 and ft3 levels, compared to GD patients without TH. As thymic involution is primarily driven by age [26], in the GD scenario also thymic enlargement can be an age-related process. Moreover, it is well known that the development of TLH is associated with several other autoimmune diseases (i.e., myasthenia gravis, systemic sclerosis, rheumatoid arthritis, systemic lupus erythematosus, pemphigus, polymyositis, and Addison’s disease) [9, 27], and this can be boosted by the coexistence of an excess of thyroid hormones in a background of thyroid autoimmunity. Yet, as reported for other extrathyroidal manifestations (i.e., GO, ALBTs, NEU) [2, 7, 8], it can be postulated also for TH that increasing thyroid hormones increase the probability of TH development. We also found in GD patients with TH a higher TgAb positivity and a lower TPOAb positivity compared to GD patients without TH [28]. Moreover, we found that abnormal ALT was more prevalent in GD patients with TH compared to GD patients without TH, which was in line with the fact that GD patients with TH also had higher ft4 and ft3 levels than GD patients without TH [7].

The pathogenesis of TH in the setting of GD is unclear but seems to involve a complex interplay of hormonal and immunological mechanisms [9]. All the 20 GD patients with TH did not have symptoms of thymic enlargement (i.e., chest discomfort, shortness of breath, pain). On the contrary, in the study by Haider et al. [9], 9 out of 13 cases of GD-associated thymic enlargement presented with symptoms of thymic enlargement, supporting that these in the context of GD do not appear to portend a higher risk for pathology warranting surgery. In GD patients, TH was larger than that found in non-GD patients and in nine out of ten cases TH disappeared after 12 months of ATD therapy with 60% of VVR at the 6-month visit. In the remaining 10% of GD cases, TH disappeared 6 months after surgery. However, also I-131 therapy can lead to involution of TH in patients with GD, as reported by Jingui et al. [29]. Imaging plays a crucial role in diagnosis and follow-up of patients with suspected thymic masses. MRI is a promising imaging modality to differentiate thymic enlargement because of high-contrast resolution and no radiation exposure. Recently, MRI has demonstrated better performance than CT for the assessment of thymic mass, especially in distinguishing thymic hyperplasia bringing out both qualitative and quantitative details [30]. To avoid ionizing radiation, especially for young patients which would undergo long periods of follow-up during therapy, we used MRI as an adequate alternative to CT. Other studies and case series demonstrated that GD-associated thymic enlargement typically regressed on radiological follow-up [9, 14, 23] providing reassurance of the benign nature of TH associated with GD. Our results are in line with these preexisting data, since they showed that the thymus regressed in all cases under euthyroidism and a high VRR of TH at the 6-month follow-up like that reported by Haider et al. [9] (i.e., 85%) was found. The benign nature of GD-associated TH was also suggested by a radiological study by Murakami et al. [23] who demonstrated a statistically significant decrease in mean thymic size and thymic density in 13 patients with GD, after 5–24 months of treatment with ATD. We found that TRAb disappearance was not necessary to have the TH disappearance, as demonstrated by the fact that in half of our cases TH totally regressed despite persistently positive TRAb levels.

In two non-GD patients with TgAb and TPOAb positivity, we detected TH. In both cases, baseline TRAb were negative, and TH disappeared after reaching normal thyroid function. Therefore, for the fact that TH can totally regress although TRAb positivity and the possibility to also encounter TH in non-GD patients with negative TRAb, we believe that in a background of thyroid autoimmunity excess thyroid hormone comes before TRAb to TH development/disappearance (i.e., reversible, and gross thymic enlargement).

There are limitations of our study that warrant some caution. First, we talked of thymic hyperplasia although we did not have anatomopathological details [i.e., true thymic hyperplasia (TTH) or thymic lymphoid hyperplasia (TLH)] [31]. However, we are confident that in all our patients with TH, thymic enlargement corresponded to a benign and hyperplastic enlargement since we demonstrated that it promptly resolved under stable euthyroidism. Second, we do not know if TH can appear again when thyrotoxicosis is not controlled, since in our patients with TH euthyroidism was stably maintained throughout follow-up. Third, perhaps we underestimated the real prevalence of TH among thyrotoxic patients, since only gross thymic enlargement which was initially seen on nUS was further assessed by thoracic MRI [31–33]. In fact, it is well known that while TTH always manifests with an enlarged thymus gland [31, 34], TLH is common histological evidence during thyroidectomy for thyrotoxicosis [32, 33]. Fourth, since we excluded GD patients with uncontrolled hyperthyroidism, we could not explore the outcome of GD-associated TH in uncontrolled hyperthyroidism. However, it is reported that failure to achieve a euthyroid state may impair resolution of the GD-associated TH [9, 14]. Fifth, we could not demonstrate the contingency of thymoma in patients with GD, since in all patients, the restoration of euthyroidism was accompanied by the disappearance of TH. However, thymoma in thyrotoxic context is very rare, considering that only three thymoma cases in anterior mediastinum and one cervical ectopic thymoma have been reported to date in patients with GD [17].

To the best of our knowledge, this is the first study allowing to calculate the prevalence of radiological GD-associated TH since it was based on a prospective cohort of unselected patients with GD. Moreover, this is the first study exploring the value of nUS as the first mean to detect TH and to reliably support the diagnosis of GD in the setting of new-onset thyrotoxicosis, also because in all cases, TH was confirmed by MRI. We cannot derive definitive conclusions regarding the debate as to whether TH represents the cause of GD or is a consequence of GD [9]. However, we believe that TH is mainly a consequence of hyperthyroidism due to GD [9, 33]. Indeed, we demonstrated that in all cases, the achievement of stable biochemical euthyrodism determined the disappearance of TH. Moreover, we demonstrated that TH also can be present in non-GD thyrotoxicosis, and it can disappear along with euthyroidism restoration. Indeed, we found the presence of TH in two non-GD cases with TgAb and TPOAb positivity, and negative TRAb: one case of silent thyroiditis, similar to the only other case report to date by Torres Gomez and Garcia Gastro [35]; instead, to the best of our knowledge, for the first time, we showed the case of a patient with thyrotoxicosis secondary to T4 + T3 therapy, where TH disappeared after switching to T4 monotherapy and achievement of biochemical euthyroidism. Therefore, as demonstrated in animals [10, 36], we postulate that administration of T3 can induce TH (i.e., TTH), and T3 levels may influence considerably thymic hyperplasia and involution.

## Conclusions

In summary, it is important for clinicians to recognize the association of TH with thyrotoxicosis for proper diagnosis and therapeutic decision-making.

We found that TH could represent a novel ultrasound manifestation of thyrotoxicosis that must be sought to accurately discriminate between GD and non-GD thyrotoxicosis. In addition, since we demonstrated that TH resolved after restoring euthyroidism, our experience supports the evidence that a conservative and non-surgical approach for the diagnostic work‐up and initial management of thyrotoxicosis-associated TH should be carefully adopted.

## Data Availability

Some or all data sets generated during and/or analyzed during the present study are not publicly available but are available from the corresponding author on reasonable request.
