# Correction: Thymic hyperplasia is accurate to detect new-onset Graves’ hyperthyroidism and resolves after restoring euthyroidism

**DOI:** 10.1007/s40618-025-02573-w

**Published:** 2025-03-25

**Authors:** L. Scappaticcio, P. Caruso, N. Di Martino, P. Ferrazzano, A. Clemente, M. I. Maiorino, A. Reginelli, G. Docimo, P. F. Rambaldi, G. Bellastella, P. Trimboli, S. Cappabianca, K. Esposito

**Affiliations:** 1 Unit of Endocrinology and Metabolic Diseases, AOU University of Campania “Luigi Vanvitelli”, Naples, 80138 Italy; 2 Department of Advanced Medical and Surgical Sciences, University of Campania “Luigi Vanvitelli”, Naples, Italy; 3 Radiology and Radiotherapy Unit, Department of Precision Medicine, University of Campania “L. Vanvitelli”, Naples, Italy; 4 Division of Thyroid Surgery, AOU University of Campania “Luigi Vanvitelli”, Naples, Italy; 5 Clinic of Endocrinology and Diabetology, Lugano and Mendrisio Regional Hospital, Ente Ospedaliero Cantonale, Bellinzona, Switzerland; 6 Faculty of Biomedical Sciences, Università della Svizzera Italiana, Lugano, Switzerland

**Correction: Journal of Endocrinological Investigation (2024) 47:2487–2497**

10.1007/s40618-025-02573-w

In the original version of this article, the author’s name Reginelli Alfonso was incorrectly written as Regginelli Alfonso.

